# Corrigendum to “The evolution of the various structures required for hearing in Latimeria and tetrapods” [IBRO Neurosci. Rep., vol. 14, June 2023, pp. 325–341]

**DOI:** 10.1016/j.ibneur.2023.04.004

**Published:** 2023-04-23

**Authors:** Bernd Fritzsch, Hans-Peter Schultze, Karen L. Elliott

**Affiliations:** a Department of Biology & Department of Otolaryngology, University of Iowa, IA, USA; b Biodiversity Institute, University of Kansas, Lawrence, KS, USA

The authors regret some amends to the wording of Supplementary Figure 1 caption were not included in the final version. The correct caption is shown below:

**Figure:**
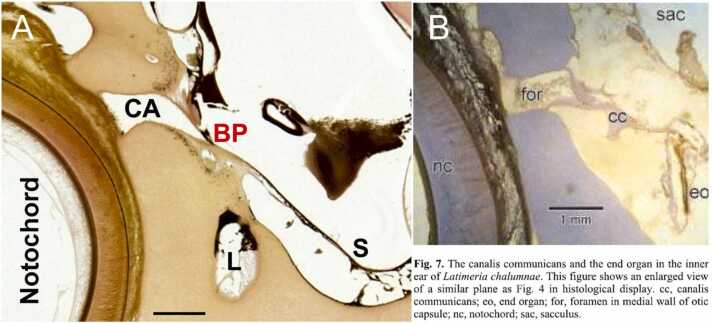

.

Supplement Figure 1.

One ear was well fixed (A) in the littermates of *Latimeria* mother that shows the cochlear aqueduct (CA) that is ending in the basilar papilla (BP). Unfortunately, the sections of Bernstein (2003) were badly fixed and lead to a different interpretation (B). First, a new end organ (eo) is suggested in a new dorsal (see Fig. 6, Bernstein, 2003) or ventral position from the canalis communicans (cc; see Fig. 7,8; Bernstein, 2003). Neither the nerve fibers nor the position that separates between the cochlear aqueduct and the basilar membrane remain unclear in the description of Bernstein (2003) that indicate a foramen (for) that ended at the BP (compare A and B). A new ‘end organ’ (B) that most likely belongs to the lagena (L) is shown below the saccule (S, see A). Not that there is no formation of a ‘canalis communicans’ that ends in the basilar papilla (compare A and B). Modified after unpublished sections (Fritzsch, 1992) and (Bernstein, 2003).

The authors would like to apologise for any inconvenience caused.
